# The Use of Lean Six Sigma for Improving Availability of and Access to Emergency Department Data to Facilitate Patient Flow

**DOI:** 10.3390/ijerph182111030

**Published:** 2021-10-20

**Authors:** Ailish Daly, Seán Paul Teeling, Marie Ward, Martin McNamara, Ciara Robinson

**Affiliations:** 1 Beacon Hospital, Sandyford, D18 AK68 Dublin, Ireland; Ciara.robinson@beaconhospital.ie; 2 UCD Centre for Interdisciplinary Research, Education & Innovation in Health Systems, School of Nursing, Midwifery & Health Systems, University College Dublin, D04 V1W8 Dublin, Ireland; sean.p.teeling@ucd.ie (S.P.T.); martin.mcnamara@ucd.ie (M.M.); 3 Centre for Person-Centred Practice Research Division of Nursing, School of Health Sciences, Queen Margaret University Drive, Queen Margaret University, Musselburgh EH21 6UU, UK; 4 Centre for Innovative Human Systems, School of Psychology, Trinity College, The University of Dublin, D02 PN40 Dublin, Ireland; marie.ward@tcd.ie

**Keywords:** emergency department, length of stay, patient flow, data analysis, Lean Six Sigma

## Abstract

The aim of this study was to redesign an emergency department [ED] data management system to improve the availability of, and access to, data to facilitate patient flow. A pre-/post-intervention design was employed using Lean Six Sigma methodology with a focus on the voice of the customer, Gemba, and 5S to identify areas for improvement in ED data management processes and to inform solutions for improved ED patient flow processes. A multidisciplinary ED team includes medical consultants and registrars, nurses, patient service staff, radiology staff, as well as information technology and hospital management staff. Lean Six Sigma [LSS] diagnostic tools identified areas for improvement in the current process for data availability and access. A set of improvements were implemented to redesign the pathway for data collection in the ED to improve data availability and access. We achieved a reduction in the time taken to access ED patient flow data from a mean of 9 min per patient pre-intervention to immediate post-intervention. This enabled faster decision-making by the ED team related to patient assessment and treatment and informed improvements in patient flow. Optimizing patient flow through a hospital’s ED is a complex task involving collaboration and participation from multiple disciplines. Through the use of LSS methodology, we improved the availability of, and fast access to, accurate, current information regarding ED patient flow. This allows ED and hospital management teams to identify and rapidly respond to actions impacting patient flow.

## 1. Introduction

The National Emergency Medicine Programme [1] advises all emergency departments [ED] to implement a six-hour standard for ED attendances so that 95% of patients are admitted or discharged within six hours of attending an ED. This target is to ensure ED patients receive timely assessment and intervention as required by their clinical presentation. This indicator aims to reduce the delays without compromising the quality of care. Inpatient boarder is a phrase used to describe a patient who has been assessed in the ED as requiring hospital admission and is waiting in the ED until an inpatient bed becomes available [1]. Prolonged delays for inpatient boarders in EDs have been shown to be associated with poorer outcomes [1]. Achieving the six-hour National Emergency Medicine Programme target requires each step of the process from the point of patient arrival and registration at the ED to their eventual discharge from the department, or their admission to an inpatient bed, to work seamlessly to ensure the highest quality care is delivered as efficiently as possible [1]. By definition, ED care is unscheduled and of varying acuity [2]. Delivery of high-quality care in an efficient manner requires clinical expertise, adequate space, and appropriate equipment, as well as timely access to meaningful data on care delivery. Clinical data supports and assists an ED team in their clinical decision making, but also, importantly, in tracking the progress of their patients’ care journey and maintaining a smooth workflow. It facilitates a better understanding of the flow of patients, bottlenecks, and patient–staff interactions [3]. The Health Information and Quality Authority states on page 4 of their 2017 “Information management standards for national health and social care data collection” document that “health information has an important role to play in healthcare planning decisions” [4]. The National Emergency Medicine Programme requires that ED information systems should be developed to facilitate measurement for ED processing times and support the delivery of high-quality care [1]. The individual time periods between each specific point of the patient’s journey are termed “turnaround time” [TAT].

The study site is an ED in a private hospital in South Dublin, Ireland. Private hospital indicates that the organization operates independently of state health services, and receives no state funding. Care is funded through private health insurance. Public health services in Ireland are provided in Health Service Executive [HSE] hospitals and public voluntary hospitals and in practice, there is very little difference between these two types of hospital [5]. Of note, many of these hospitals also provide private health care but they must clearly distinguish between public and private beds.

All Irish residents are eligible for public healthcare; however, there are noted variations in coverage, access, and cost, depending on a person’s income, geographic location, and the length of time it takes to receive care. Currently, 45% of the population has voluntary health insurance [6]. In Ireland, a ten-year plan to achieve universal healthcare “Sláintecare” was published in 2017, with the action plan launched in 2019, and this is currently ongoing.

In the study site, the patient journey through the ED commences with registering for the service with patient service staff. This triggers a process by which the patient interacts with multiple healthcare staff across all grades and disciplines and the process includes:

◦ completion of a nurse-led triage assessment;
◦ completion of physician and nursing assessments;
◦ completion of diagnostics such as radiology and pathology that inform clinical decisions regarding admission as an inpatient or discharge to outpatient care.

In the study site, the patient may have direct or indirect contact with up to fifteen different staff members across up to five different hospital departments. Target turnaround times for triage and completion of assessment as demonstrated in Table 1 are based on the Manchester Triage System [7], a system of clinical risk management employed in EDs worldwide to manage patient flow safely when clinical need exceeds capacity. It sets the target times by which patients assessed in different categories of severity should be seen [7].

Other aspects of the ED pathway are context-specific and subject to local arrangements; for example, the TAT from referral to specific diagnostics to receipt of results, and the TAT from the decision to admit to actual patient admissions to an inpatient bed. Therefore, optimizing patient flow through ED is multifactorial [8] with many points of data entry, access, and collection by staff. Optimizing patient flow is dependent on the ability of multidisciplinary teams to meet the needs of the acutely unwell patient and on the capacity within a hospital to triage, assess, admit, and discharge patients. Importantly, given the multiple steps in the process, it is critical to success to meet the ED staff requirement for easily accessible, relevant data to monitor a patient’s journey, comply with targets, and identify areas for improvement in patient flow [9]. This study discusses a process redesign to improve the availability of, and ED staff access to, relevant data to facilitate patient flow.

The setting for this study was a consultant-led ED in a private hospital in Dublin with a capacity to see and treat 55 patients per day. Private hospital indicates the organization operates independently of state health services and receives no state funding. Care is funded through private health insurance. Patients may self-present or be referred to the ED by their general practitioner. The ED does not accept ambulance admissions and it does not offer 24-h cover, operating between 10:00 and 19:00. This is an important difference in the arrangements of EDs in public hospitals in Ireland. Patients must be admitted or discharged each evening; there is no option for patients to remain in the ED overnight. An initial verbal inquiry to the ED staff as to issues that affected patient flow indicated that access to data was considered to be the primary factor and they found the current process to be slow and cumbersome. This access to data to inform patient flow was, and is, important as ED patient flow impacts the entire hospital system. Longer lengths of stay [LOS] in the ED affects many stakeholders as follows: the patient, in terms of potential delays in care and treatment; admitting teams, as admission assessments are required later in the evening or overnight when staffing is reduced, and ED staff, as overtime is required if the patient’s care in the ED has not been completed by the end of the scheduled shift [10]. There were 180 h of ED nursing overtime in January and February 2020, leading to concerns within the ED and wider management team about staff wellbeing, which included occupation-related fatigue [11]. Finally, any delay in ED patient flow and LOS has a corresponding effect on bed requirements for scheduled surgery within the operating room [OR]. Patient feedback on their ED experience was also considered. However, the main suggestions for change from patients were related to invoicing for their ED visits and did not reference their actual care needs. In December 2019, the hospital executive management team [EMT] identified improving patient flow as an area for targeted improvement. As the first step in this improvement process, and reflective of staff feedback, consistent accurate data regarding ED patient flow would be required. In keeping with the hospital’s strategic approach to process improvement, Lean Six Sigma [LSS] was the improvement methodology of choice. LSS methodology has been effective in reducing LOS in a hospital ED [9,10]; Futera and colleagues [12] highlighted access to information technology and data-driven improvement as key facilitators for change. This process improvement, therefore, aimed to improve the availability of, and ease of access to, ED patient flow data management through the application of LSS methodology.

LSS has been used in healthcare since the early 2000s to improve efficiency and achieve quality and operational excellence [13]. Since healthcare providers worldwide, whether publicly or privately funded, are faced with similar challenges of caring for an aging population with a limited pool of financial and personnel resources, the need to seek efficiencies while continuing to provide quality services has become more and more acute [14]. LSS has been implemented in many healthcare organizations with improvements achieved across many clinical and administrative pathways and processes, including medication management, specific patient conditions such as stroke [15], OR organization and efficiency [16], and appointment and clinic management [17]. Lean or LSS has been utilized in EDs to improve waiting times and patient flow [18].

The hospital adopted LSS as a methodology for process improvement in 2017. By 2020 the LSS programme in the organization had matured to a team of 13 advanced improvement practitioners from disciplines including nursing, physiotherapy, speech and language therapy, administration, and patient services who had completed a post-graduate certificate or diploma training in LSS Process Improvement in Healthcare. These practitioners had previously delivered process improvement projects across a wide variety of topics including streamlining of booking of elective surgeries, reducing LOS in elective orthopedic surgery, as well as procurement and theatre stock management. The University training programme carried out with the study site’s academic partner University College Dublin [UCD], aims to give staff an appreciation of systems and to avoid using LSS as a decontextualized toolkit [19].

## 2. Methods

A pre-post study design measures a variable of interest before and after an intervention with the same location, setting, and participants [20]. For this study, a pre-/post-intervention design was employed using Lean Six Sigma methodology to measure variables related to the availability of and access to ED data within the ED setting and with specified participants [ED doctor, nurse, and patient service team members]. The design enabled us to measure the impact of a LSS redesign of existing processes for data access and retrieval within the ED. The LSS Define, Measure, Analyze, Improve, Control [DMAIC] framework was utilized to structure the improvement. The LSS tools used throughout the improvement process are set out in Table 2.

### 2.1. Define

A LSS project team, convened by a graduate of the University education and training programme, was established to carry out this process improvement, as outlined in Table 3. The support of the EMT facilitated engagement from the emergency department and supporting teams. Having a data analyst/information technology specialist on the team was crucial to providing detailed statistics behind ED processes, which was a challenge to the completion of the project as it ran during the organization’s response to the first wave of COVID-19 in Ireland. IT resources were reassigned to implement urgent technical solutions as part of the organization’s response to the COVID-19 pandemic.

DMAIC provided a model for the structured approach set out in a project charter [21]. A team project charter was agreed upon with the support of the CEO and deputy CEO with a project goal of having timely access to accurate information about ED patient flow for both the immediate ED team and associated departments, as well as for the EMT. The project goal was SMART [22], i.e., there was a specific goal, the achievement of which would be measurable through the hospital’s electronic patient record [EPR] system and business intelligence system. The target was deemed to be achievable with engagement from all relevant stakeholders and was considered to be relevant as it aligned to hospital data management strategy. The project timeline was set to start November 2019 for completion July 2020.

Baseline departmental data at the point of commencing the project indicated:

◦ In 2019, the ED received 45–55 presentations each day.
◦ ED visits increased from 8773 in 2017 to 10,100 in 2018, and 11,186 in 2019.
◦ The percentage of patients admitted to an inpatient bed was 32% in 2017, 29% in 2018, and 27% in 2019.
◦ The median LOS in the ED in the period 2017–2019 was 3 h 35 min; however, 16% of patients had a LOS of which exceeded 6 h in that period.
◦ There was no single source for data regarding ED patient flow. The ED management team accessed three different data reports and four different sections of the electronic patient record for information regarding patient flow.

**Figure 1:**
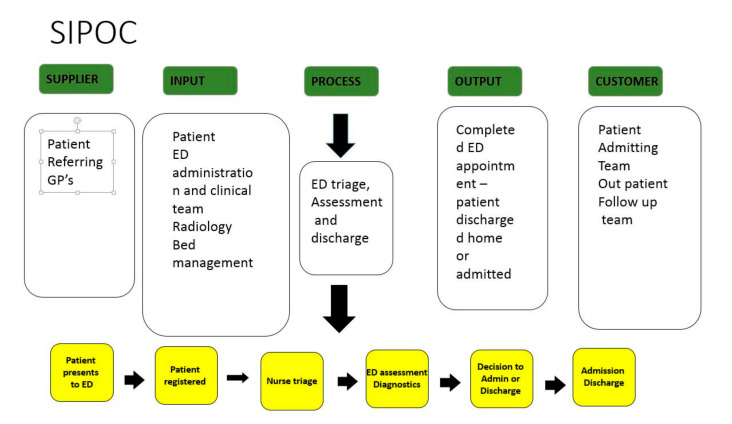
SIPOC.

Stakeholder engagement was informed by person-centered collaborative, inclusive, and participatory [CIP] principles [31,32] which have been shown to be synergistic with LSS use in healthcare [15,33,34]. In practice, the project group sought active participation and input from stakeholders in defining a minimum dataset required to make visible the ED patient flow. This dataset was informed by the experts on the ground involved in delivering care rather than defined by a remote management team. The stakeholders were also instrumental in discussing the ideal process for mining and presenting data. Having the IT analyst participate as a key stakeholder from the outset ensured that when decisions were taken concerning data required, the process for extracting this data was assessed and confirmed as achievable/not achievable from the outset. A communication plan was implemented with ongoing stakeholder engagement sessions with all key participants including ED teams, radiology, the EMT, inpatient admitting teams, and with information technology staff included from the outset. The first output from the stakeholder engagement sessions was a high-level process map or SIPOC [21] of visits to the ED [Figure 1]. The SIPOC enabled us to visualize the variables involved in the process and facilitated all stakeholders having a clear, visual reference for what inputs were required in the process to facilitate required outputs to satisfy both patients and staff.

### 2.2. Measure

**Figure 2:**
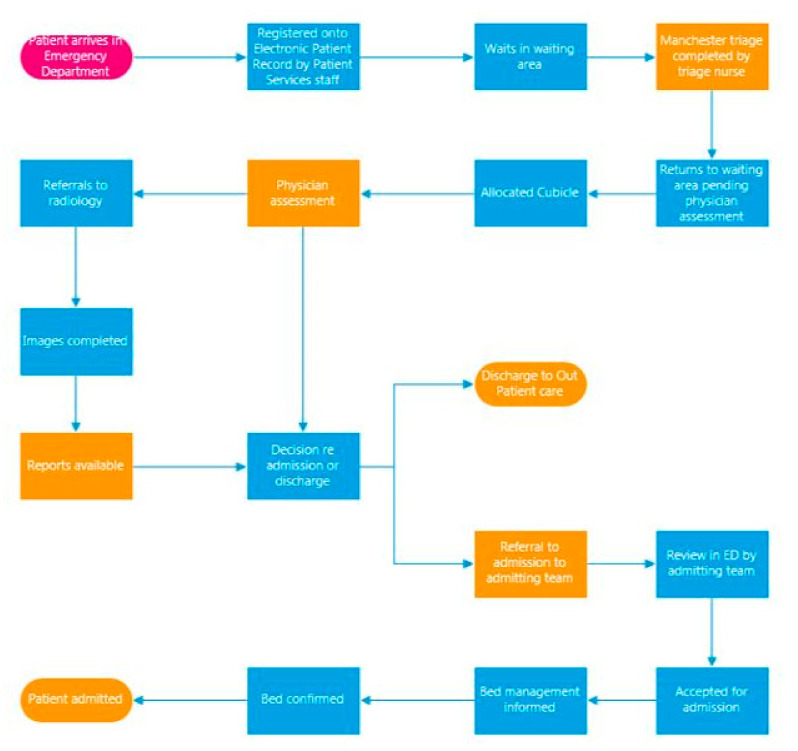
Process map of emergency department patient flow.

The terminology “voice of the customer” [VOC] is used in Six Sigma to denote the expectations of the customer; in healthcare, the customer can be staff or patients or any participant in the delivery or receipt of care [26]. Following five VOC sessions, and building on the SIPOC, a more detailed process map [Figure 2] was developed. The process map was used to highlight areas where data access and availability had a particular impact on patient care and correspondingly patient flow. Six critical areas, highlighted in orange in Figure 2, indicated these areas.

The process map was validated by the ED team who worked in and with the process on a day-to-day basis. The process map further facilitated discussion and led to a more detailed capture of the VOC. Bertels [25] discussed how VOC data is gathered, and then mapped onto a LSS tool known as a critical to quality [CTQ] tool. A CTQ tool is designed to capture the key measurable characteristics of a process or service whose performance standards must be met to satisfy the customer. A CTQ tool was completed. The CTQ characteristics of the ED patient flow process were identified as follows:

1. Ease of access to and time taken to access ED patient flow data;
2. ED LOS;
3. TAT for completion of nurse triage, physician assessment, and completion of radiology.

It was clear from our discussion with the full project team that LOS and TAT [characteristics 2 and 3] were influenced by staff access to real-time data [characteristic 1]. Combining knowledge gained from the SIPOC exercise and CTQ allowed the creation of a data collection plan. Ease of, and timely, access to data was identified as a primary outcome. Processes impacting on patient flow that were influenced by data availability were broken down to achievement of ED triage time and achievement of assessment targets.

A Gemba walk is an observation/understanding of where and how the work is done and is an important component of the LSS approach [27]. Gemba were completed by observing the journey of the patient through the ED. Accompanying audits were completed through data mining from the hospital’s EPR system enabling data such as registration time, triage time, physician assessment time, and LOS to be recorded. ED operations were altered dramatically during the COVID-19 lockdown period as the department changed from a walk-in service to telephone triage and appointment-only service for the period 16 March through to 1 August 2020. Gemba and audits were repeated during this period [June 2020] and again in August 2020.

**Data availability:** To source complete datasets regarding the patient flow for one patient required access to four separate clinical records on the EPR. The staff user had to click in and out of four sections of the patient record, including consultant records, medical records reports [which include non-consultant hospital doctors reports], diagnostic imaging reports, and assessment forms [which include nursing records]. Three different data reports were involved, an ED nursing report, ED doctor report, and ED patient experience time report. Data access to inform decision-making was observed as taking an average of 9 min per patient (*n* = 45). With the ED averaging 45 patients per day, collating this data takes between 5 and 6 hours of ED staff time.

Table 4 shows the ED patient volumes and acuity, as well as key turnaround times.

**Achievement of targets:** In August 2020, a Gemba of patient flow within the ED confirmed the following median times:

◦ From arrival in the ED to triage was 17 min;
◦ To physician assessment was 38 min;
◦ TAT to completion of radiology was 2 h, 5 min;
◦ Median LOS was 3 h, 43 min [well below the NEMP 6 h target].

**Special cause variation:** Lessons learned during the period of the COVID-19 lockdown included:

◦ LOS targets were not achieved for 10% of patients attending the ED.
◦ Access to inpatient beds was also not a limiting factor during the COVID-19 lockdown period as bed occupancy was at 54%.
◦ Access to radiology reports was not a limiting factor in this period as radiology was completed immediately on request. The instantaneous availability of radiology during COVID-19 lockdown was due to general outpatient radiology activity being significantly reduced resulting in the ED having almost exclusive access to radiology resources, in effect special cause variation [35].

The COVID-19 lockdown period taught us some valuable lessons–improving bed availability and access to radiology in isolation would not guarantee a reduction in ED LOS without the ability to improve patient flow within the ED itself, which as outlined was impacted by the availability of data.

### 2.3. Analyze

A failure mode effect analysis [FMEA] and fishbone cause-effect analysis were completed. An FMEA is a product risk assessment that analytically approaches the prevention of defects by prioritizing potential problems and their resolution [28]. The FMEA identified the completion of the ED-based processes including triage and physician assessment, as well as completion of radiology processes as a high risk both in occurrence and detection with scores of 225, 360, and 360, respectively. These scores indicate that there is a high chance of target times for these processes not being met as well as a high chance that deficiencies in these TAT will be undetected.

**Figure 3:**
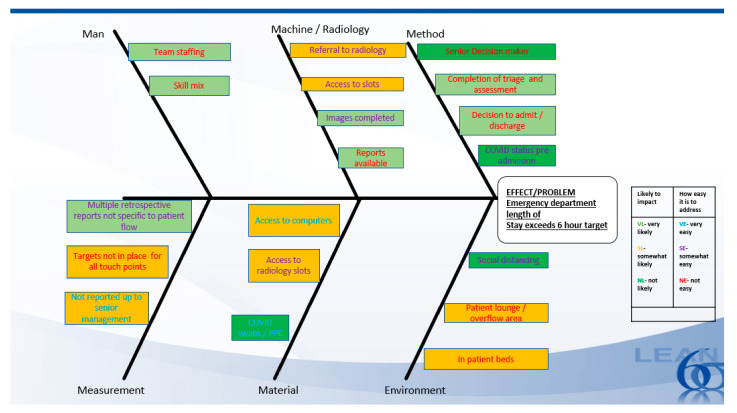
Fishbone cause-effect analysis.

Doggett [36] (2005) wrote that cause-effect analysis diagrams illustrate the possible causes of a particular problem by sorting and relating them using a classification scheme. In this project, the fishbone cause-effect analysis as demonstrated in Figure 3 supported further insight into probable causes of ED TAT not being detected which then had an impact on patient flow.

### 2.4. Improve

Following analysis, a brainstorming session with the project team agreed to first focus improvements on the measurement of the process. It was agreed that without immediate access to accurate data the impact of further improvements would be difficult to assess. Once accurate, timely data was available, processes such as admission to inpatient bed could be examined and targeted improvements implemented.

#### Data Availability

Stakeholder engagement sessions were conducted to co-create the required data set for the ED and method for presentation. As described earlier, the ED data was available in various forms and reports. It was agreed to utilize a 5s approach to conclude the final dataset. A 5s is a popular tool within the lean paradigm, for organizing spaces so work can be performed efficiently, effectively, and safely. While 5s is intended for a physical work environment, its central function is to organize, standardize, and maintain through visual management [21] which we translated for use in analyzing the current process for data access [Table 5].

The 5s exercise (Table 5) was used to illustrate the current state and target states and led to agreement on the following improvements:

◦ Daily ED patient flow report.
◦ Reduce from 7 ED patient data sources to 1 report.
◦ Reduce from 73 general to 37 data points specific to patient flow.
◦ Available at a set time each day, no data mining is required.
◦ On the advice of the IT analyst, it was agreed to complete the daily tracker as a first step. The IT build required for a “live tracker” would be extensive. The project team agreed to assess the impact of the daily tracker, re-confirm the minimum dataset, and then proceed to the live tracker.
◦ Governance structure agreed–report available to ED team including the ED clinical nurse manager, ED nurse coordinator, ED consultant on duty, and ED patient services lead. Availability of this data is important to the ED team in order to monitor patient flow daily and identify and guide improvements. If targets are not met, the ED management is aware immediately and implement timely interventions. The ED management also has data available to share with wider stakeholders to inform wider process change and improvements, for example, negotiate increased access to radiology and guide improvements in the admission process. The hospital EMT utilizes data to inform strategic planning for the ED, for example, data availability regarding patient acuity [demonstrated by Manchester score] informs the need for increased senior decisionmaker staffing; referrals to radiology inform decisions regarding expanding of radiology support and services to the ED.
◦ Obstacles to the achievement of targets can be managed in a proactive rather than reactive manner.

In addition to the above key improvements, some quick wins were also identified, for example, more bedside computers were purchased to allow for bedside completion of electronic radiology referrals/radiology reports, and ED/radiology operations team meet weekly to identify and agree on the need for extra ED specific slots.

### 2.5. Control

A control plan was devised to support and monitor continued improvements. The impact of the availability of ED patient flow data was monitored through stakeholder feedback, monitoring compliance with turnaround targets as well as informing strategic decisions. Achievement of the ED patient flow targets was reassessed in March, May, and August 2021.

## 3. Results

**Figure 4:**
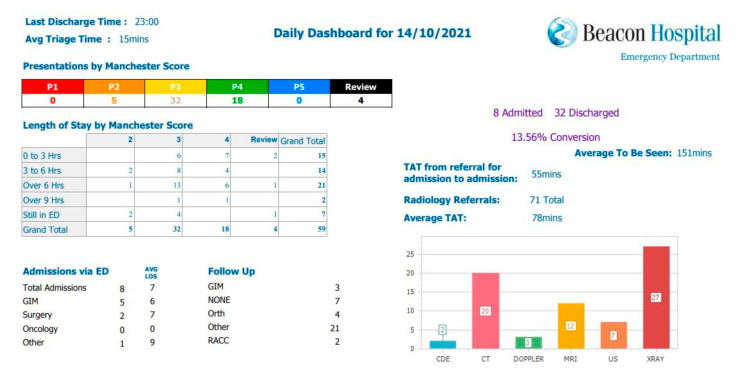
Sample of the ED daily report.

One organization-wide ED activity report is now circulated to the EMT and the ED team each morning at 9 am as per sample in Figure 4. Key metrics including patient volumes, Manchester score, LOS exceeding 6 and 9 h, achievement of triage and assessment targets, and radiology volumes are captured in this report. The report has reduced time spent compiling patient flow reports from 9 min per patient to 0 min. At the commencement of this project, the ED department saw from 45 to 55 patients per day. This equated to 405–495 min of nursing time occupied with compiling reports which now have been reduced to zero minutes. The report is immediately available. Reducing time spent on data management releases the ED nurse manager time for other duties including patient care, staff support, as well as service improvement and development. Availability of accurate relevant data allows the ED team to identify areas for improvement in patient flow.

### Data Available Shows

◦ In terms of activity, the number of ED presentations increased monthly during the control period from 929 presentations in March 2021 to 1154 presentations in August 2021.
◦ Patient acuity was largely unchanged, the majority of patient presentations categorized into Manchester score 3, requiring urgent but not immediate care.
◦ Median time to completion of triage, assessment, and LOS increased during the control phase as a consequence of increased patient presentations [Table 6]. Time to completion of assessment and length of stay remains within the National Emergency Medicine Programme targets of 3 h to completion of assessment and 6 h for LOS. Time to triage falls outside the National Emergency Medicine Programme target of 15 min.

## 4. Discussion

By definition, an ED is unpredictable. Optimizing patient flow is dependent on processes within and outside the ED operating efficiently and effectively. The main learnings from this improvement are as follows:

- There is no single factor upon which ED LOS will succeed or fail.
- Proactive/on-the-spot visibility over the process, through data, information, and knowledge sharing, is essential for optimizing patient flow.

### 4.1. Factors That Influence ED LOS

#### The Importance of Sharing Data, Information, and Knowledge

Availability of the ED daily report allowed the stakeholders to gain an accurate insight into the needs and challenges which confront the ED daily. The ED clinical nurse manager no longer has to spend 9 min per patient data mining to establish bottlenecks in patient flow. This information is immediately available to the ED team and also to members of the EMT. VOC feedback from improved reporting include:

“The new report is great, when I see triage and assessment times are slipping, I can follow it up immediately with the team member” [ED clinical nurse manager].

“Tell me about triage scoring–can we use it to better predict admission requirements for ED patients” [commercial director].

Availability of this information has helped inform decisions regarding inpatient bed allocation including earmarking specific beds to the medical admissions unit.

Healthcare is frequently described as fragmented or siloed, and this is reflected in how data is captured, managed, and shared throughout the system. Ward et al. [37] noted that data relating to business performance, quality, and patient safety is extracted from different systems, and its primary use is to inform senior decision-makers about organizational-level performance rather than to support those at the front line in understanding and improving their daily performance. It is estimated that up to 30% of the total health budget may be spent on handling data and information, i.e., collecting it, looking for it, and storing it [38]. This study identified significant resources dedicated to handling data; however, the process for translating that data to meaningful information regarding patient flow and making that information available to frontline staff is onerous and time-consuming, limiting the knowledge gained related to the process.

Data regarding ED patient flow must be translated into accessible knowledge and ultimately wisdom, as Ackoff outlined in his classic data, information, knowledge, understanding, wisdom hierarchy [39]. This knowledge and wisdom can drive performance improvement at the team/unit level. At the commencement of this process improvement, we collected data which was occasionally processed by an individual and disseminated at the time of crisis. As the process improvement enters the control phase, we now use the data in an organized team manner to create meaningful knowledge about the ED processes. Following Ackoffs theory, this will further evolve into a shared understanding between the ED team and the EMT which will guide further ED process improvements. Strome (2013) described the challenge of information overload and the need to harness data to improve clinical and organizational performance [40]. This process improvement is a first step in harnessing data re ED patient flow. The availability of timely, relevant, accurate, complete, valid data [4] regarding ED patient flow has given both the local ED and the EMT knowledge regarding ED patient flow and has helped inform decision making regarding ED operations; a key function of data gathering in Ackoff’s theory. The next step is to make this data available in real-time which will allow immediate interventions when challenges to ED patient flow arise.

### 4.2. Systems Issues

At the commencement of this process improvement, VOC and stakeholder engagement sessions pointed at perceived bottlenecks in the system such as access to radiology as well as inpatient beds as main areas to address. Lessons learned during the COVID-19 lockdown prompted the project team to investigate further and gain more knowledge from the data available. We avoided the temptation to jump to immediate conclusions [such as add radiology slots or reserve inpatient beds for patients awaiting admission from ED]. Instead, we took an “outside-in” perspective. We recognized the need to see the data regarding ED patient flow from an external perspective and utilize knowledge gained to co-create solutions to challenges identified [41].

Future areas to focus on, include the following:

- Process:
  ◦ Access to data regarding referrals for admission per specialty will give insight into the potential benefits of the system-wide “fast track” admission process to specialties, for example, assess the impact of suggested medical admission pathway prior to reporting of radiology [42,43].
  ◦ Access to data regarding outpatient follow-up requirements per specialty will inform the requirement to reserve appointments for outpatient follow-ups as an alternative to admission.
  ◦ In this area of improvement, we will employ a more complex interdepartmental application of lean, as we utilize the voice of the customer across admitting and outpatient teams, observe the process for inpatient admissions, as well as outpatient follow-ups and work with stakeholders to implement change [43].
- Staff/team working
  ◦ A second-generation project will examine the potential role of advanced nurse practitioners in EDs. Advanced nurse practitioners have been established in public EDs with a proven impact on improving patient flow and delivery of care [44]. The knowledge regarding ED patient flow made available from this project will give the second-generation team a platform to examine what tasks could be shared from the ED consultant and non-consultant hospital doctor team to advanced nurse practitioner, potentially improving the TAT to completion of the ED assessment, and therefore improving LOS.

An appreciation of the system [43], in which inquiries are conducted and improvements implemented, was critical to the combined and effective use of the person-centred and LSS improvement approaches we undertook. As well as contributing to the use of LSS in access to and use of LSS, we feel this study contributes to the wider body of knowledge in the use of LSS and person-centred approaches, an under-researched area [45,46].

### 4.3. Strengths and Limitations

The evolving LSS culture in the hospital supported a system-wide approach to improving access to ED data. Rather than working in isolation, the ED team worked across silos involving patient services, radiology, information technology, and EMT, as well as the lean practitioner in analyzing the process and formulating solutions.

It is recognized that busy hospital staffs often work in departmental silos and do not see the entire service [46,47]. However, LSS can facilitate breaking down these barriers to facilitate a system vision or perspective. According to Graban (2012, p. 1).

“Lean is an approach that can support employees and physicians, eliminating roadblocks and allowing them to focus on providing care. Lean helps break down barriers between disconnected departmental ‘silos,’ allowing different hospital departments to better work together for the benefit of patients”.

The strengths of this project were the stakeholder involvement. Changing from what could be construed as a silo approach to analyzing ED performance to involving all stakeholders in the process. The EMT sponsorship supported the project team in suggesting change. The EMT participated actively in contributing to stakeholder engagement sessions regarding minimum dataset/data requirements.

This process improvement is one part of a wider improvement plan to reduce ED length of stay which is ongoing. This study, however, has given a solid platform to understanding ED patient flow. We can now analyze the system factors and relationships around ED patient flow and implement informed solutions.

## 5. Conclusions

Optimizing patient flow through EDs is a key target for any healthcare organization. Identifying and addressing challenges in isolation is unlikely to lead to success. ED patient flow is multifactorial. An understanding of the challenges and opportunities for improvement at each stage of the process is essential. Through adapting a LSS approach including cross-disciplinary stakeholder engagement, rigorous data analysis, and person/process centered improvements, we have begun the process for improving LOS in hospital ED. System vision and awareness and person-centered approaches contributed to a wider understanding of the factors involved. Ongoing control and monitoring of this improvement will be required which will also identify further avenues for improvement within and outside the ED. This will contribute to the ongoing development of a lean culture in the organization.

## Figures and Tables

**Table 1:** Manchester Triage.

Category	Priority	Maximum Time [min]
1	Immediate	0
2	Very Urgent	10–15
3	Urgent	60
4	Standard	120
5	Not urgent	240

**Table 2:** LSS Tools.

Title of Improvement Tool	Definition	Output
Project charter [21]	A Project Charter is used to define, act on, and review challenges and problems.	It was useful in clearly identifying the goals of the project as what was in scope.
Developing SMART goals [22]	Goals that are specific, measurable, achievable, relevant, and time-bound.	SMART goals agreed.
SIPOC [21]	A high-level view of the process with SIPOC standing for suppliers, inputs, processes, outputs, and customers.	Identify linkages between suppliers, customers, inputs, outputs, and process.
RACI [23]	Describes essential roles and responsibilities of each member on a project or task. Responsible, accountable, consulted and informed.	Ensured all stakeholders were involved and engaged throughout the process improvement.
Stakeholder meetings [24]	Consultation with the stakeholders to establish the problem, expectations, and potential causes and to create solutions.	
CTQ [25]	Critical to quality tree: The CTQ tool is designed to capture the key measurable characteristics of a process or service whose performance standards must be met to satisfy the customer.	Critical to quality metrics identified: length of stay, the turnaround time for completion of triage, assessment, diagnostics, and decision to admit. Data availability for each metric
VOC [26]	Voice of the customer: what is the customer looking for?	Identified needs of the customer-patient, ED team, and organization.
Gemba [27]	Observation/understanding of where and how the work is done.	Understand the ED process and identify potential bottlenecks.
Fishbone [28]	Fishbone diagrams are used during brainstorming to enable root cause analyses.	Targeted areas for improvement.
FMEA [28]	Failure mode and effect analysis is a risk analysis tool that is used to prevent an event from happening. It highlights the aspects of a process that should be targeted for improvement.	Prioritizes/highlights the aspects of the process that should be targeted for improvement.
Process map [29]	Process mapping [PM] supports a better understanding of complex systems and adaptation of improvement interventions to their local context.	Agree on the as-is process and opportunities for understanding bottlenecks.
5s [21]	Creates the visual workplace: sort, set in order, shine, standardize, sustain.	Agree on data set for emergency department patient flow.
Control plan [30]	What was measured, why, who is responsible, and action required.	Agree on monitoring and progressing improvements.

**Table 3:** Project team.

Position	Project Role	Expertise
Chief executive officer/executive management team [EMT]	Exec sponsor	Commercial and strategic ED targets
ED clinical nurse manager	Process owner	ED process flow, key inputs/outputs
ED lead consultant	Stakeholder	Clinical oversight for ED patient care
Head of radiology	Stakeholder	Radiology referral and report process
Patient services manager	Stakeholder	First point of contact for patient Data collection
IT analyst	Stakeholder	Data analysis and management
Lean Six Sigma practitioner	Project owner	LSS process improvement, limited knowledge on ED process–provided a fresh perspective on the process

**Table 4:** Activity, patient acuity, patient flow data for the emergency department January, June, and August 2020.

	January 2020	June 2020	August 2020
Total ED presentations	1029	532	773
Manchester scores [%]			
1	0%	0%	0
2	11%	8%	14%
3	46%	50%	43%
4	38%	31%	35%
5	0%	0%	1%
Manchester score blank	5%	11%	7%
Median time to triage [hh:mm:ss]	00:17:00	00:00:00	00:17:00
Median time to physician assessment	00:32:00	00:35:00	00:38:00
Median time to completion of radiology	01:40:00	00:01:00	02:05:09
Median LOS	03:50:00	03:20:00	03:43:00
LOS > 6 h [%]	22%	10%	17%
LOS > 9 h [%]	2%	2%	1%
ED admissions	13%	30%	20%
Occupancy	91%	54%	72%

**Table 5:** 5S.

5s	Current State	Desired Future State
Sort	7 Sources ED datasets 9 min per patient, retrospectively	1 All-inclusive source report available, live in the ED tracker, 9:00 a.m. each morning for the previous day
Set in order	73 Raw data points TAT calculated manually	Data points specific to patient volumes and flow Average turnaround time calculated
Shine	Raw data presented in an inconsistent format, not color-coded	Example: Time formatted [1] in minutes only Color coding for Manchester scoring Target for physician assessment versus Manchester score
Standardize	Metrics presented person dependent	Standard daily report with comparable data
Sustain	No consistent data reporting	Daily report circulated to the executive management team and ED team

**Table 6:** Activity, patient acuity, patient flow data for the emergency department control phase–March, May, and August 2021.

	March 2021	May 2021	August 2021
Total ED presentations	929	1012	1154
Manchester scores [%]			
1	0%	0%	0
2	17%	16%	12%
3	44%	48%	48%
4	38%	36%	38%
5	0%	0%	0%
			
Median time to triage [hh:mm: ss]	00:17:00	00:18:00	00:24:00
Median time to physician assessment	01:08:00	01:00:00	01:25:00
Median Length of stay	03:57:00	04:12:00	04:25:00
LOS > 6 h [%]	16%	21%	13%
LOS > 9 h [%]	1%	1%	1%
